# Assessment of Micro-Basin Tillage as a Soil and Water Conservation Practice in the Black Soil Region of Northeast China

**DOI:** 10.1371/journal.pone.0152313

**Published:** 2016-03-31

**Authors:** Yuanyuan Sui, Yang Ou, Baixing Yan, Xiaohong Xu, Alain N. Rousseau, Yu Zhang

**Affiliations:** 1 Key Laboratory of Wetland Ecology and Environment, Northeast Institute of Geography and Agroecology, Chinese Academy of Sciences, Changchun 130102, China; 2 Graduate University of Chinese Academy of Science, Beijing 100049, China; 3 Soil and Water Conservation Research Institute of Jilin Province, Changchun 130033, China; 4 Centre Eau Terre Environnement, Institut National de la Recherche Scientifique (INRS-ETE), 490 de la Couronne, Québec, Qc, Canada; DOE Pacific Northwest National Laboratory, UNITED STATES

## Abstract

Micro-basin tillage is a soil and water conservation practice that requires building individual earth blocks along furrows. In this study, plot experiments were conducted to assess the efficiency of micro-basin tillage on sloping croplands between 2012 and 2013 (5°and 7°). The conceptual, optimal, block interval model was used to design micro-basins which are meant to capture the maximum amount of water per unit area. Results indicated that when compared to the up-down slope tillage, micro-basin tillage could increase soil water content and maize yield by about 45% and 17%, and reduce runoff, sediment and nutrients loads by about 63%, 96% and 86%, respectively. Meanwhile, micro-basin tillage could reduce the peak runoff rates and delay the initial runoff-yielding time. In addition, micro-basin tillage with the optimal block interval proved to be the best one among all treatments with different intervals. Compared with treatments of other block intervals, the optimal block interval treatments increased soil moisture by around 10% and reduced runoff rate by around 15%. In general, micro-basin tillage with optimal block interval represents an effective soil and water conservation practice for sloping farmland of the black soil region.

## Introduction

Soil erosion is one of the most serious global environmental problems threatening food security and human sustainable development [1]. The Hei Longjiang Province, Jilin Province, Liaoning Province and the eastern Inner Mongolian Province, the renown Mollisols (black soil) regions of Northeastern China, produce 17.1% of the nationwide total grain yield [2]. However, according to a government report (Ministry of Water Resources, 2002), about 27.59 ×10^4^ km^2^ of this region is prone to significant soil and water losses (accounting for 27% of total areas), where 46.53% of the agricultural land slopes are less than 7°, yet the range of slope lengths ranges from 200 to 1000 m [3]. Sloping croplands have become the primary source of soil erosion in the black soil region of Northeastern China [4]. Serious soil erosion induces thin soil layers, soil structure damages and productivity losses, threatening the precious black soil resource [2,5,6]. Although many best management practices, such as conservation tillage, terraces, strip cultivation, crop rotations, have been developed, given land occupation and high implementation cost, these practices are not readily accessible to the local people [3].

Micro-basin tillage is a soil and water conservation practice characterized by individual earth blocks built along furrows [7–9]. It has been widely used in different places and known under different names such as furrow-diking, diked furrows, tied ridges, basin tillage and basin listing [10,11]. Studies have suggested that micro-basins could enhance infiltration, increase rainfall use efficiency and soil water content, and improve crop yield [10–13]. For example, Jones and Clark [14] examined the potential of furrow dikes to retain surface runoff in the Southern Great Plains of USA and found that reduction of 25 to 30 mm of runoff could be achieved on an annual basis with maximum runoff retention up to 111 mm. In Northern China, for sloping land with gradients of 5%, 10%, 15% and 20%, Xiao et al. [15] reported that micro-basins were cost-effective measures decreasing runoff and soil loss by 70% and 62%, respectively. In Africa, Sanders et al. [16] inferred that tied-ridging could increase agricultural economic benefits by 12%. In the southern United States of America, Nuti et al. [9] stated that furrow diking significantly reduced water consumption and increased on average cotton yield by 171 kg/ha.

Many factors influence the effectiveness of micro-basin tillage as a soil and water conservation practice, including natural factors and anthropogenic factors, such as rainfall, topography, soil properties, design parameters and tillage methods [7,17]. In the black soil regions of China, given furrow-ridge tillage has construction standards, the volume of surface flow captured by a micro-basin depends on the block interval length, which governs the water storage capacity per unit area [18]. With a longer interval, the effectiveness of micro-basins could be sharply decreased. However, a too-short interval will not only reduce runoff retention, but will also steeply increase implementation cost [19]. Thus, farmers will have little incentive to apply this technique in their agricultural practices [20]. In fact, since most studies only assessed the effect of basin tillage with random block interval lengths, the results have not been deemed robust and somewhat misleading for farmers or local agricultural agencies.

Therefore, there is a need to conduct a study on the efficiency of micro-basin tillage with respect to surface flow retention using the optimal block interval length algorithm [21]. To facilitate the experimental process and data collection, rainfall simulators are widely applied in soil erosion studies [15,22,23]. However, when conducting field experiments, simulated rainfall becomes vulnerable to meteorological conditions (speed and wind direction), and thus, the monitored data (runoff and sediment) are always different from those obtained under natural rainfall conditions [24]. For more robust conclusions, it becomes necessary to collect data from field standard plots under natural precipitations. Most of the past studies focused on the impact of micro-basin tillage with respect to water and soil control, but few studies attempted to assess this practice in terms of nutrient losses [15,17]. Some research results showed that micro-basin tillage was an effective water quality best management practice (BMP) and could increase grain yield [11,16,17]. If this technique could significantly reduce agricultural nonpoint pollution from sloping land, local governments and farmers would have an incentive to apply micro-basin tillage in black soil regions of Northeastern China.

The goal of this study was to determine the influence of the block interval length on the efficiency of micro-basin tillage applied in sloping lands of the Black Soil Region of Northeast China. The specific objectives were to: (1) assess the effects of micro-basins on soil water content; (2) identify impacts of micro-basins on controlling water and soil erosion losses; and (3) evaluate the influences of micro-basins in reducing diffuse pollution and increasing grain yield.

## Methods and Materials

The granted permission of this project was from Soil and Water Conservation Research Institute of Jilin Province, China. The National Park was a public place for the researchers, so there were no specific permissions for related studies. In addition, the field studies did not involve endangered or protected species.

### 2.1 Study area

**Fig 1:**
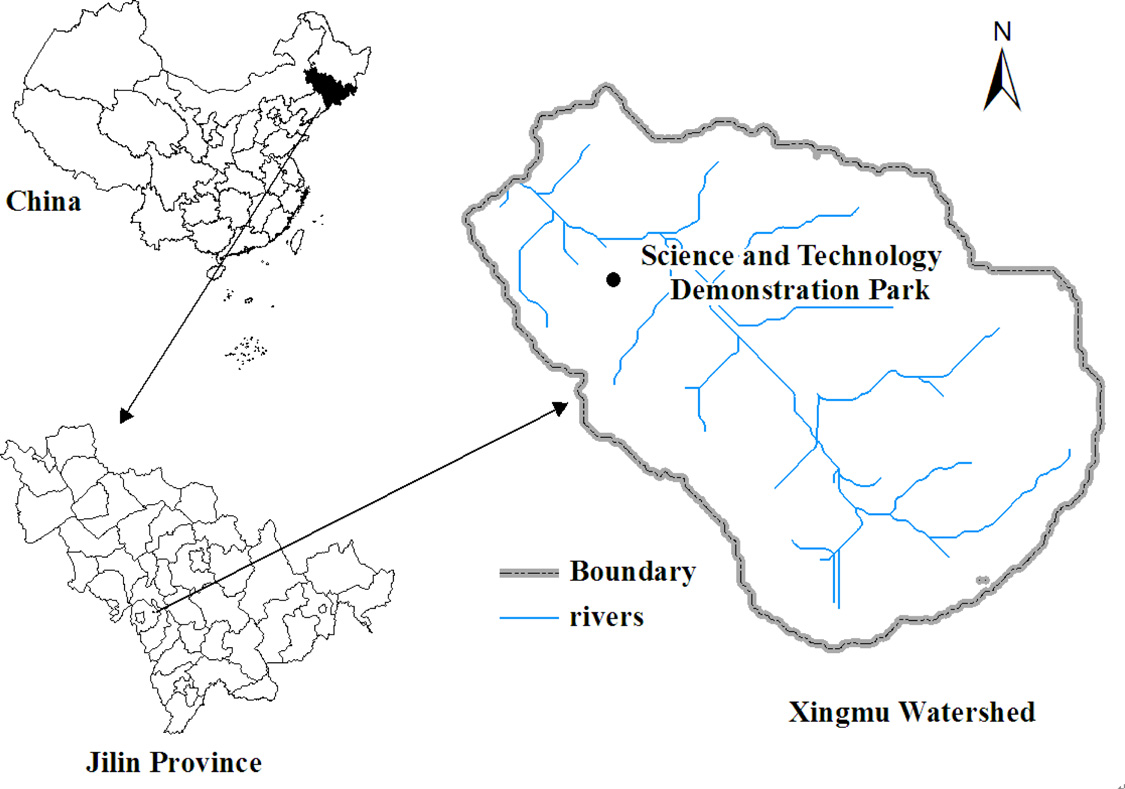
Location of the study area in Xingmu watershed.

This research was conducted at the National Soil and Water Conservation Science and Technology Demonstration Park in Xingmu watershed, Dongliao County, Jilin Province, P. R. of China (E 125° 22' N, 42° 58'), Fig 1. The study area is located in low mountains and hills of the black soil regions of Northeast China. It can be considered as a cold temperate zone, semi-humid and continental monsoon climate. The annual average temperature is 5.2°C and the annual average precipitation is 658 mm, with 80% of the total rainfall occurring between June and August [25]. The major economic crop is maize. The main soils are black soils and brown soils. The soil chemical properties of the first 20 cm and the 20–40 cm of soils at the experimental site are introduced in Table 1. Due to serious soil erosion, the soil organic matter level of the study area was much less than the average level (more or less 20 g kg^-1^) in the Jilin Province.

**Table 1:** Soil chemical properties as a function of depth at the experimental site.

Soil property	0–20 cm	20–40 cm
organic matter (g kg^-1^)	10.58	10.13
Total nitrogen (g kg^-1^)	0.83	0.51
Total phosphorus (g kg^-1^)	0.61	0.46

### 2.2 Optimal block interval model

**Fig 2:**
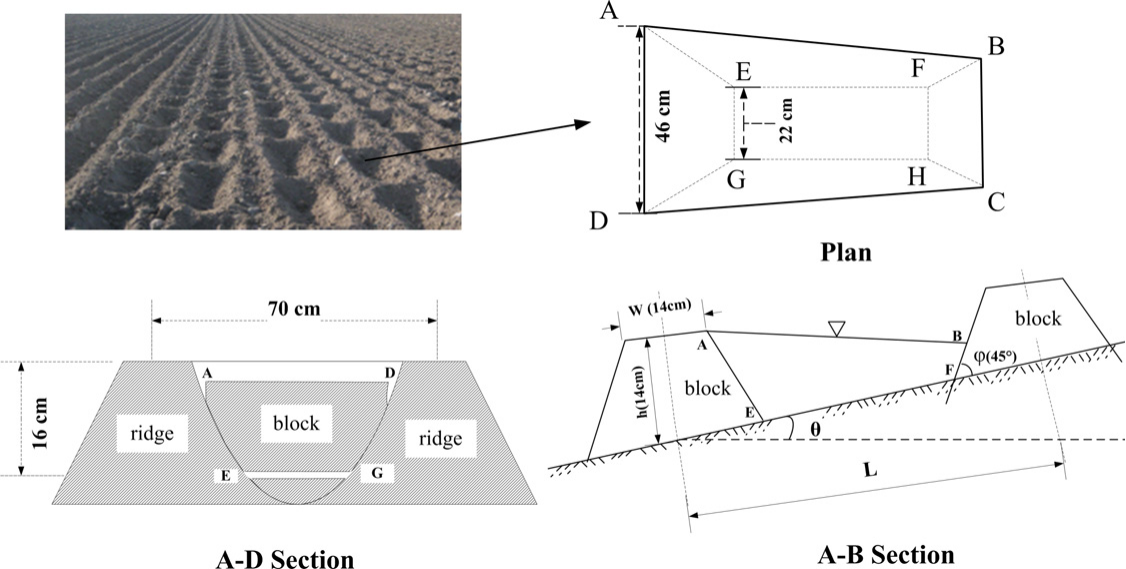
The multi-view of a model micro-basin.

The algorithm of the optimal block interval was established based on the structural parameters of the conventional ridge with respect to capturing maximum runoff volume per unit area [3]. According to field surveys of the geometry of the ridge-furrow widely applied in black soil regions of Northeast China, it was found that the spacing and height of the ridge were 70 cm and 16 cm, respectively. The water storage volume of micro-basin was estimated to be similar to that of a quadrangular frustum pyramid (Fig 2).

Using the three-dimensional geometric analysis unveiled by [2,20], a single micro-basin volume model was deduced as follows:

$$\begin{array}{l} V=\frac{h}{6}[LW+(L-2ctg\varphi \cdot h)\times (W-2ctg\varphi \cdot h)+4(L-ctg\varphi \cdot h)\times (W-ctg\varphi \cdot h)]\\ -\frac{1}{4}L^{2}W(\text{sin}\;2\theta -2ctg(\theta +\varphi ){\text{sin}}^{2}\;\theta )+\frac{1}{6}L^{3}ctg\varphi {\left(\text{sin}\;2\theta -2ctg(\theta +\varphi ){\text{sin}}^{2}\;\theta \right)}^{2} \end{array}$$ (1)

V—volume of micro-basin (cm^3^); θ—slope gradient along the ridge (°); φ—slope gradient of block (°); L—block interval length (cm); h—height of block (cm); W—width of block (cm).The height(h)and width (W) of the block are both 14 cm in the field, the unit ridge length is defined as constant (L_1_). Thus, the runoff retention volume unit ridge length(V_1_)was calculated using Eq (2) as follows:

$$\begin{array}{l} V_{1}=\frac{V\times L_{1}}{L}=\frac{5000h}{3L}[LW+(L-2ctg\varphi \cdot h)\times (W-2ctg\varphi \cdot h)+4(L-ctg\varphi \cdot h)\times (W-ctg\varphi \cdot h)]\\ -2500LW(\text{sin}\;2\theta -2ctg(\theta +\varphi ){\text{sin}}^{2}\;\theta )+\frac{5000}{3}L^{2}ctg\varphi {\left(\text{sin}\;2\theta -2ctg(\theta +\varphi ){\text{sin}}^{2}\;\theta \right)}^{2} \end{array}$$ (2)

L_1_ –unit ridge length (10000 cm defined in this study)

After a thorough analysis of Eq (2), it was found that there was a nonlinear relationship between L and V_1_ with respect to each gradient (0.5°≤θ≤25°), so the algorithm of L threshold (optimal block interval-L_2_) corresponding to the maximum runoff retention volume (V_max_) was:

$$L_{2}=168\theta^{-0.5}$$ (3)

### 2.3 Experimental design

**Fig 3:**
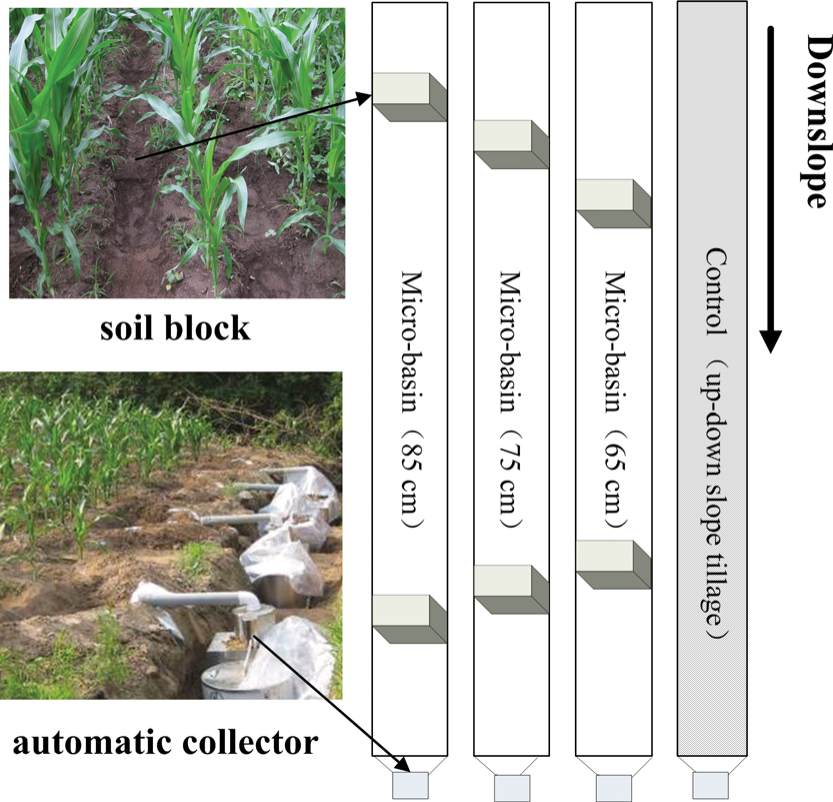
The sketch map of the micro-basin tillage.

The experimental work was conducted in 2012–2013. It was based on two independent variables that included the block space (block interval length) and slope gradient. In the black soil regions of Northeast China, 80% of agricultural land has a slope gradient smaller than 7°, twelve plots (4 treatments ×3 replications) were established on 5° sloping land in 2012 and twelve plots were installed on 7° sloping land in 2013. According to Eq (3), treatments of control and micro-basins with block space of 65 cm, 75 cm (optimal block interval for a 5°-slope) and 85 cm were built in 2012. Treatments of control and micro-basins with block space of 53 cm, 63 cm (optimal block interval for a 7°-slope) and 73 cm were installed in 2013. Each plot, 25-m long and 1.8-m wide, had three ridges. Maize (*Zea mays*), a major food crop in the study area, was planted using traditional cultural practices on May 1^st^ of each year. A sketch map of the experimental plots is shown in Fig 3. In order to moderate the impact of micro-basins on operation of farm machinery, the basins were built after the maize elongation stage (more or less June 12).

In this study, soil water content was measured by time domain reflectometry (TDR) [18]. In 2012, because the instrument was hit by thunder, the monitoring periods were from July 19 to July 26 and August 19 to August 26. In 2013, the monitoring periods included two months (July and August). Monitoring started at 9:30 am every day. Portable runoff automatic collection devices were placed in the center of three ridges at the bottom of each plot [26]. After each rainfall, 500 ml runoff with sediments was collected and then refrigerated within 24h for analysis. The concentrations of total phosphorus (TP), total nitrogen (TN) and sediments were measured based on national standard methods [27]. There were 26 and 23 rainfall events monitored in 2012 and 2013, respectively, 80% of which occurred during the growing season (June-August). Meanwhile, given relatively large differences in rainfall amounts and rainfall intensities among the three months (Table 2), runoff and sediments were categorized on a monthly basis. In addition, four whole rainfall-runoff processes were recorded in 2012 (August 2 and 19) and 2013 (July 13 and August 16). Maize yield was assessed by harvesting total crop of each treatment plot [8].

**Table 2:** Characteristics of rainfall during growing season.

Year	Index	June	July	August
2012	Precipitation (mm)	121	211	156
	Average rainfall intensity (mm/hr)	14	18	10
2013	Precipitation (mm)	100	181	99
	Average rainfall intensity(mm/hr)	11	15	8

#### 2.4 Data analysis

The experimental data were processed using analysis of variance (ANOVA) and least significant difference (LSD) among control and micro-basin tillage with three different block spaces. The test results of the treatment effects were considered significant at *p* = 0.05. SPSS 16.0 (SPSS, Inc., USA) was used for all the statistics. The data was deposited in the supporting information database (S1–S6 Files). All the representation and graphs of the monitored data were illustrated with OriginPro 8.0 (Originlab, Inc., USA).

## Results

### 3.1 Effects of micro-basin on soil moisture

**Fig 4:**
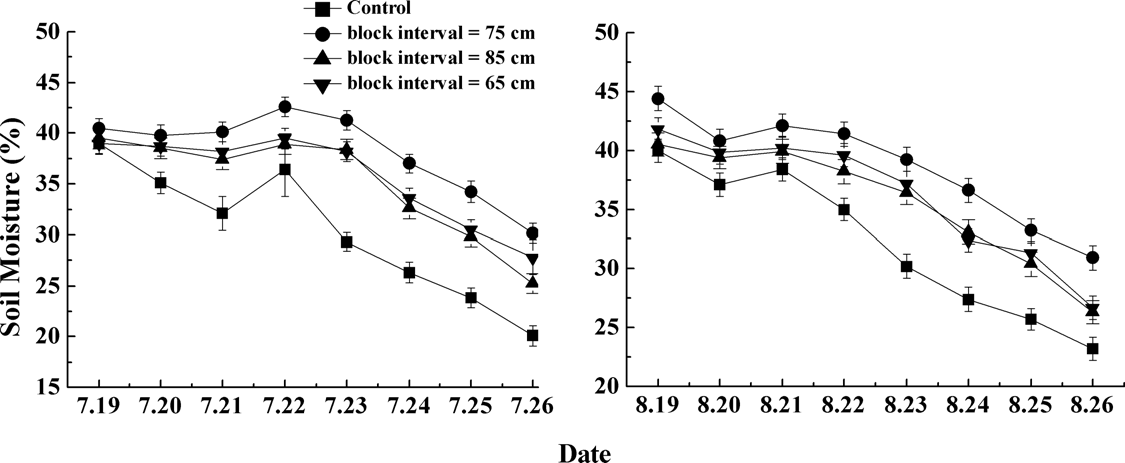
Temporal variation of soil water content in 2012 (slope = 5°). Note: Error bars represent one standard deviation.

The soil water content was continuously measured during 8 days in each month of July and August. As shown in Fig 4, it can be concluded that micro-basin tillage had a significant positive effect on soil water retention in sloping land. From July 19 to 26, the soil water content over a depth of 0–20 cm of the control plot went down from 39% to 20%. When compared to the control plot, the average soil water content of micro-basin tillage treatment plots increased by 29% (85 cm), 40% (75cm) and 34% (65cm), respectively. Meanwhile, Fig 4 shows that the soil water content of the control plot was the lowest among all the four treatments all the time, but that of the basin tillage treatment plots with 75-cm block space were the highest all the time and increased up to 50% on July 26. The treatments of each 65- and 85-cm block spaces had almost the same impact on increasing soil water content. Similarly, during August 19–26, compared with other treatments, basin tillage with 75-cm block space had the highest soil water content (on average 39%). When compared to the control plot, the soil moisture of the micro-basin tillage plots, with 65-cm, 75-cm and 85-cm block spaces, increased by 12%, 20% and 11%, respectively. These results imply that micro-basins tillage could effectively improve soil water content of sloping land. Moreover, monitoring data of the micro-basin treatments with 75-cm block space (for slope gradient of 5°) were in agreement with the calculation results of the optimal block space algorithm.

**Fig 5:**
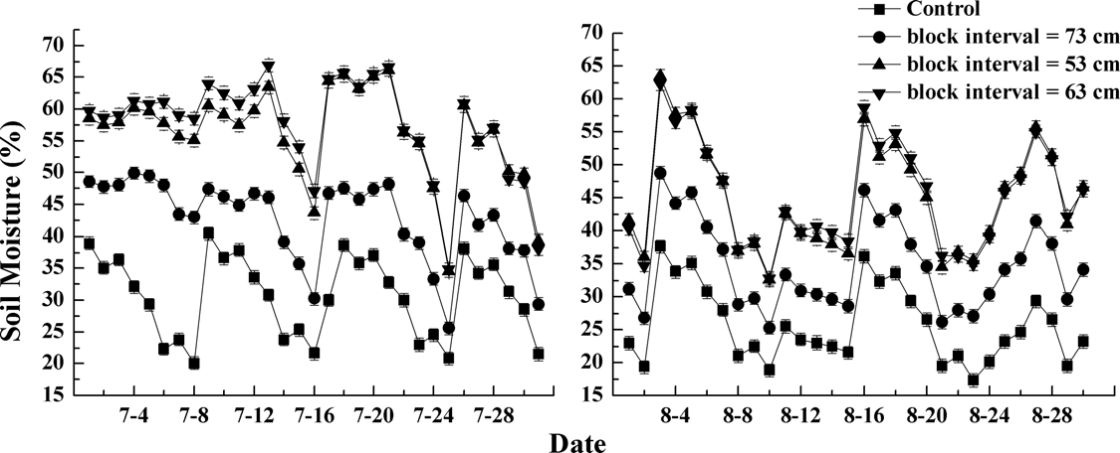
Temporal variation of soil water content in 2013 (slope = 7°). Note: Error bars represent one standard deviation.

Fig 5 shows that soil water content over the first 20 cm of soil of all the micro-basin treatment plots (7°-slope) were larger than that of the control during the two months of 2013. In July, soil water content ranged from 21% to 40% in the control. As before, the 63-cm block space treatment, designed using the optimal algorithm, has the largest positive impact on soil water moisture, reaching values up to an 88% increase with respect to the control. The 53-cm and 63-cm block space treatments had a merely equal effect on improving water storage capacity. The former treatment increased soil water content by 76%, while in the latter treatment, the efficiency increase was only 1% more than that in former one.

### 3.2 Effects of micro-basin tillage on runoff

**Fig 6:**
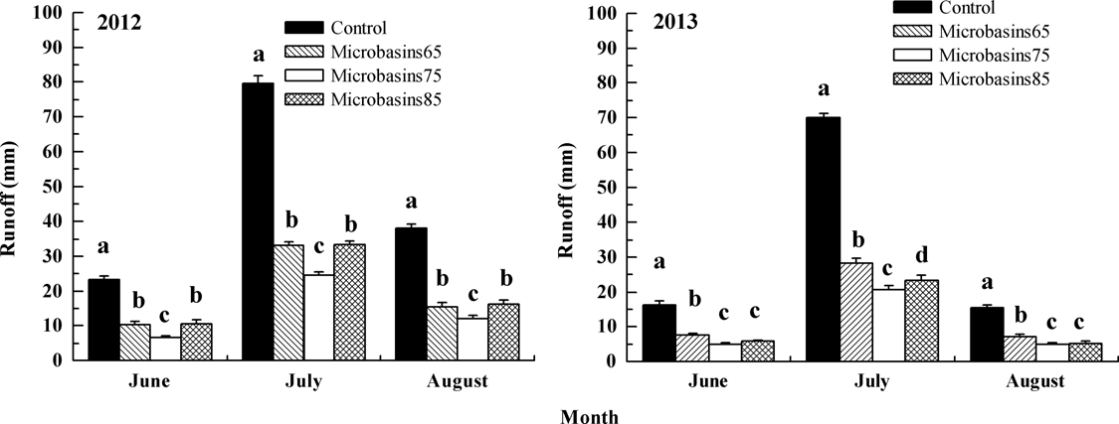
Runoff of the different treatment plots under two sloping land gradients. Note: Different levels (a, b, c and d) presented within the same month show that the values were significantly different at the 5% probability level. Error bars represent one standard deviation.

Fig 6 indicates that in 2012 and 2013, micro-basin tillage mitigated on average runoff by 61% and 64%, respectively. In 2012, micro-basins with 75-cm block space (5°-slope) had the least amount of runoff, which were 7 mm, 25 mm and 12 mm from June to August, respectively. While compared with the control, micro-basin tillage with different block spaces reduced runoff by 69% to 71% (75-cm block space), 56% to 59% (65-cm block space) and 55% to 58% (85-cm block space). According to ANOVA results introduced in Fig 6, the differences in runoff between control and three micro-basin tillage plots were significant (*df* = 11, *F* = 147.45, *p* < 0.01 for June; *df* = 11, *F* = 904.07, *p* < 0.01 for July; and *df* = 11, *F* = 318.46, *p* < 0.01 for August); in addition, the differences in runoff between 75-cm block space and the other two block spaces were also significant (*p* < 0.01 for June, July and August); however, the differences between treatments of 65-cm and 85-cm block spaces were not significant all the time (*p* >0.10 for June, July and August).

As seen in Fig 6, in 2013, micro-basin treatments on 7°-sloping land mitigated runoff by 68% to 71% (63-cm block space), 65% to 67% (53-cm block space) and 54% to 60% (73-cm block space) compared to the control. The ANOVA results in Fig 6 shows significant differences in runoff between the control and micro-basin treatments (*df* = 11, *F* = 175.60, *p* < 0.01 for June; *df* = 11, *F* = 851.93, *p* < 0.01 for July; and *df* = 11, *F* = 137.90, *p* < 0.01 for August). Meanwhile, there was a significant difference in runoff between micro-basins with 63-cm block space and that with 73-cm block space (*p* < 0.01 for June, July and August). However, no significant differences were found between micro-basins with 63-cm block space and that with-53cm block space (*p* > 0.10 for June and August), except in July (*p* = 0.03).

### 3.3 Effects of micro-basin on rainfall-runoff process

**Fig 7:**
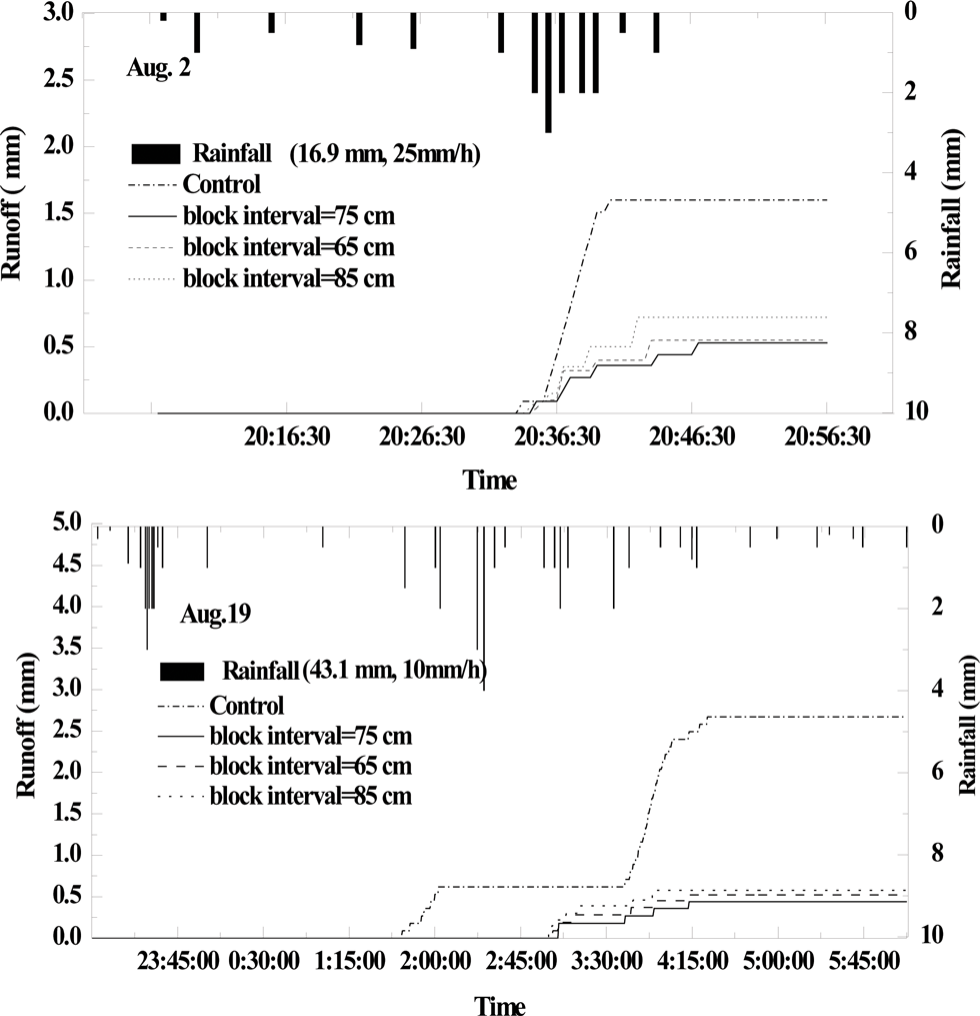
Rainfall-runoff process of different treatments for two rainfall events of 2012.

There were only two whole rainfall events recorded in 2012. The first one occurred on August 2^nd^, the amount and intensity were 16.9 mm and 25 mm/h respectively. As seen in Fig 7, the total runoff (1.6 mm) of the control was much more than that of the micro-basin treatments, which were 0.55 mm (65-cm block space), 0.53 mm (75-cm block space) and 0.72 mm (75-cm block space). Meanwhile, the initial runoff generation time of the micro-basin plots was relatively later than that of the control, that is a significant portion of runoff was captured in the micro-basin. Runoff increased gradually and slowly in the micro-basin tillage treatment plots during the rainfall event, especially in the 75-cm block space. The second rainfall occurred on August 19th and precipitation and rainfall intensity were 43.1 mm and 10 mm/h. Runoff of the control was still the largest with 2.67 mm for the whole rainfall event. Although the average runoff reduction rate of the three micro-basin treatments was higher than 78%, the differences among them were small. In addition, compared to monitoring data of August 2nd, the micro-basin tillage had more influence on holding off water and, thus, increasing the initial runoff generation time.

**Fig 8:**
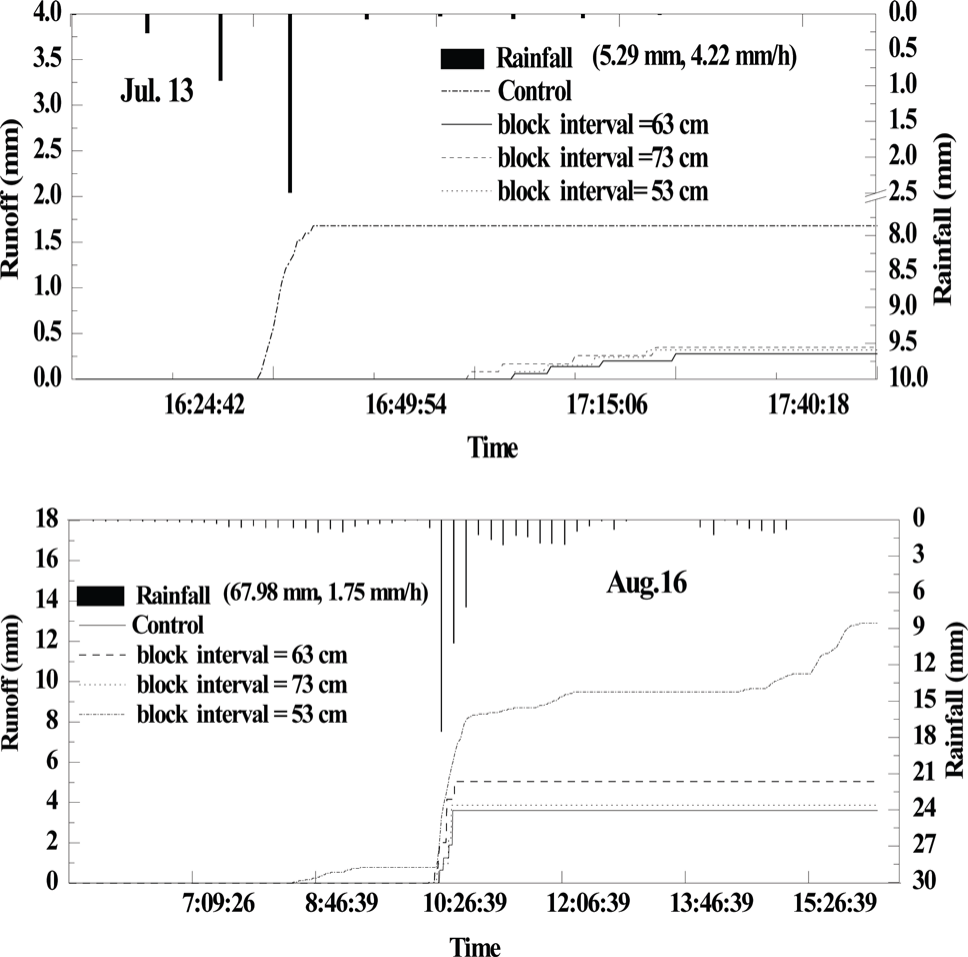
Rainfall-runoff process of different treatment plots under two rainfall events of 2013.

The monitored data of July 13, 2013 (Fig 8) show that the control not only produced earlier runoff, but also had the greatest total runoff (1.68 mm). The average runoff reduction rate of the micro-basin treatments was 80%. Similarly, for August 16, runoff of the control was much larger than that of the micro-basin treatments. Runoff mitigation rate of micro-basin tillage was more or less 72%, and the initial runoff generation time was significantly longer during the rainfall events. Moreover, the differences among the micro-basin treatments were larger than those of July 13.

### 3.4 Effect of micro-basin tillage on soil erosion

**Fig 9:**
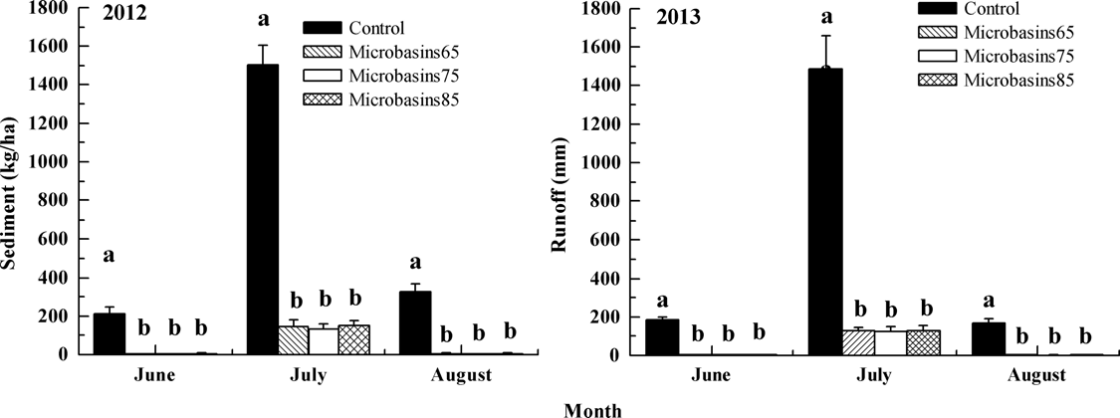
Sediment loss of the different treatment plots under two sloping land gradients. Note: Different levels (a, b and c) presented within the same month show that the values were significantly different at the 5% probability level. Error bars represent one standard deviation.

Sediment loss over the two years was summed up for each treatment (Fig 9). The results indicated that the quantity of sediment increased dramatically with increasing rainfall amounts; however, the increase was remarkably lower in all micro-basin treatment plots with respect to the control. It could be attributed to the fact that runoff from control had a larger output rate and would continue for a longer period, increasing sediment loss. When compared to the control, sediment reduction rates of the micro-basin treatments were all more than 90% under two sloping land gradients during the two years; corresponding better abatement rates than those for runoff reduction. According to ANOVA results introduced in Fig 9, differences in sediment loss between control and the micro-basin tillage plots were significant for both years; that is in 2012 (*df* = 11, *F* = 142.38, *p* < 0.01 for June; *df* = 11, *F* = 424.56, *p* < 0.01 for July; and *df* = 11, *F* = 204.05, *p* < 0.01 for August) and in 2013 (*df* = 11, *F* = 441.01, *p* < 0.01 for June; *df* = 11, *F* = 170.32, *p* < 0.01 for July; and *df* = 11, *F* = 169.27, *p* < 0.01 for August). However, the difference of influence on soil erosion between the optimal block-space treatments and the other two block space treatments was not significant in 2012 (*p* > 0.10 for June, July, August) and 2013 (*p* > 0.10 for June, July, August).

### 3.5 Effect of micro-basin tillage on nutrient losses

As seen in Table 3, micro-basin tillage had large positive impacts on nutrient loads. For 2012, when compared to the control, micro-basin plots reduced TN loads by 86% (65-cm block space), 90% (75-cm block space) and 86% (85-cm block space). The reduction rates of TP loads were 80%, 86% and 80% respectively. Although nutrient loads were a little less for the 7°-slope in 2013 than that in 2012, the reduction rate increased by around 5%. The micro-basins with 75-cm and 63-cm block space still had the largest mitigation rates (*i*.*e*., for the two sloping land gradients).

**Table 3:** Nutrient loads of the different treatments under two slope gradients.

Nutrient	Sloping land gradient (5°) 2012				Sloping land gradient (7°)2013			
	Control	Micro-basins(65cm)	Micro-basins(75cm)	Micro-basins(85cm)	Control	Micro-basins(53cm)	Micro-basins(63cm)	Micro-basins(73cm)
TN(kg/ha)	19.07±0.33	2.67±0.15	1.92±0.19	2.76±0.11	13.35±1.22	1.50±0.07	1.25±0.04	1.89±0.08
TP(kg/ha)	4.44±0.29	0.87±0.07	0.61±0.07	0.90±0.03	3.18±0.10	0.37±0.01	0.32±0.02	0.49±0.03

### 3.6 Effect of micro-basin tillage on maize yield

As reported in Table 4, all micro-basin treatment plots were characterized by increased crop yields during the two years. When compared to the control in 2012 and 2013, average increase in annual rate was about 17% for both years. The two treatments with optimal block space (75 cm and 63 cm) had both the largest yields under the two sloping land gradients.

**Table 4:** Crop yields of different treatments.

Year	Treatment	Block space (cm)	Annual yield (kg/ha)	Increase rate (%)
2012	Micro-basins	65	9120±382	17
		75	9315±473	18
		85	9211±529	16
	Control		7893±354	
2013	Micro-basins	53	9300±517	16
		63	9433±632	18
		73	9247±672	16
	Control		7985±380	

## Discussion

Recent research demonstrated that soil moisture with micro-basin tillage was around 50% more than with control treatments [22], so for this study area, this technique could be considered as an effective practice for soil erosion control and water conservation. The efficiency of micro-basin tillage was higher in 2013 than in 2012, and we propose two main reasons for this result. First, the efficiency of basin tillage appeared to vary with respect to rainfall amount. Because rainfall was 29% more in 2012 than in 2013, the saturation period could have been longer in the former year. Thus in 2012, the difference in soil moisture was relatively minor between control and micro-basin tillage. Second, slope affected the soil water content. In general, surface runoff was positively correlated with the slope, which shortened the infiltration time. So, compared to the data obtained for a 5°slope in 2012, soil water content with micro-basin tillage was larger than that of the control for a 7°-slope in 2013, resulting in an increased efficiency. According to the 2013 results, treatments with block spaces of 63 and 53 cm had a better effect on improving soil water content (Fig 5). According to Figs 4 and 5, the observed trends illustrate that the optimal block space algorithm substantially improved the determination of the optimal spacing and, thus, the efficiency of the micro-basin tillage practice. However in 2013, the 7°-sloping plots, with the optimal block space (63 cm) treatment had almost an equal impact on soil moisture than the 53-cm block space treatment. It could be deduced that the water retention volume unit area was a key factor in the design of the micro-basin tillage.

Micro-basin tillage had a relatively high runoff mitigating efficiency in this study. The results were in agreement with the conclusion reported in many previous studies [28–30] which showed that soil-block micro-ponds dramatically mitigated runoff and increased infiltration [18]. In terms of runoff volume, the runoff reduction rate of all micro-basin treatment plots was positively correlated with rainfall amount. In July, due to the largest rainfall amount of the whole year, the efficiency was at a maximum value under the two slope conditions. However, compared to the results for the 5°slope plots, the difference in runoff volume between the optimal block treatment (63 cm) and the 53-cm block treatment was minor. Because surface runoff was positively related with the slope, the probability of runoff generation in optimal block treatment dramatically increased, declining the efficiency of the optimal block design for the 7°-sloping land. From the runoff generation process point of view, with respect to control, micro-basin tillage not only mitigated the peak runoff rates, but also delayed the beginning of the runoff generation process [15,17]. Nevertheless, rainfall intensity had a relatively large effect on water retention under micro-basin tillage conditions [23]. Thus, initial runoff generation time would become similar to that of the control once the rainfall intensity was more than a certain threshold. Therefore, in region with high rainfall intensity, some other BMPs, such as vegetative filters, should be used in combination with basin tillage.

The soil loss was effectively controlled by micro-basin tillage. It could be attributed to the fact that micro-dams (soil block) built with the basin tillage, forming a relatively large surface roughness, capturing more water and increasing duration of time for lateral and vertical infiltration, can reduce the kinetic energy responsible for detachment and transport of soil particles [10,19]. The concentration of runoff into rill represents a key driving factor of soil erosion. In addition, during 2012 and 2013, the sediment reduction rate (around 90%) of all the micro-basins treatments was more in this study than that (40%–80%) reported in other related studies [18,22]. One of the main reasons was the depth of the top soil layer in the study area was similar to that of the plow pan. This was due to serious soil erosion over the past 30 years (1980–2012), so sediment loss was relatively small in the control, ranked in the lowest class according to Standards for classification and gradation of soil erosion in China (SL190-2007) [2]. The rainfall intensity was another important factor, 75% of which was lower than 25 mm/h, the erosive rainfall threshold for the study area. In addition, the high sediment mitigation rate also resulted in minor differences of soil loss among the micro-basin tillage treatments with the three block intervals. Due to the thin soil layer and small rainfall intensity, sediment yield was relatively small, so all of the three micro-basin tillage treatments could intercept most of it during the whole rainy season.

Runoff and sediments are both vital driving factors of nutrient loads [9,21,28]. Given that micro-basin tillage substantially reduced runoff rate and sediment loss, it should not be a surprise that nutrient loads would be reduced accordingly [22,28,29]. Reduction in nutrient loads was a little more under micro-basin tillage than those for water loss and soil erosion control practices currently applied in the study area [3,5]. When compared to the 2012 results, TN and TP loads of all treatments significantly declined due to lower rainfall amount in 2013. In addition, TP reduction rate under micro-basin tillage conditions was a little lower than those for TN. It could be explained by the facts that there was a relatively large difference in soil absorption mechanism between phosphorus and nitrogen [31]. The phosphorus is usually absorbed to sediments, reducing phosphorus infiltration in soils [32]. Meanwhile, small losses of sediments (Fig 9) also affect the phosphorus mitigation efficiency of the basin tillage.

In general, maize yield was the worst under up-down slope tillage (control). Indeed, it was around 85% of that for micro-basin planting. The increase in maize yield for the plots under micro-basin tillage was analogous to that of the land with cattle manure, indicating that micro-basin represents a high benefit-cost ratio solution [2,3,6]. The results showed that basin tillage significantly increased soil water content and reduced sediment and nutrient losses by increasing infiltration time and soil roughness during the growing season, while increasing mean grain yield in the study area. However, there are many other factors that can influence crop growth and yield, including crop characteristics, climate conditions, soil characteristics and human activities [33]. In this study, micro-basin tillage greatly improved soil moisture conditions, significantly exceeding plant requirements. Meanwhile, many research suggested that maize is a kind of drought-tolerant crop [34]. Thus, difference of the three micro-basin tillage treatments, following the variation of soil water content, could not result in remarkable difference in yields among different treatments.

## Conclusions

This study evaluated the efficiency of micro-basin tillage with different block spaces on sloping land of Northeastern China. The conclusions are as follows:

Micro-basin tillage had a dramatic impact on increasing soil moisture and maize yield, reducing runoff, sediment and nutrient loads during the growing season. Meanwhile, micro-basin tillage could reduce peak runoff rates and also delay initial runoff-yielding time on associated with the rainfall-runoff process.

The optimal block interval algorithm proved to be important for improving the efficiency of micro-basin tillage for soil water conservation and nutrient loads mitigation. Compared to the other two block interval treatments, the optimal block interval treatments increased soil moisture by around 10% and reduced runoff rate by around 15%. However, from a sediment loss control and yield increase point of view, optimal block interval had relatively small influence on micro-basin tillage due to meteorological factor and soil property.

Overall, micro-basin tillage, with optimal block interval, proved to be an effective practice to control water loss, soil erosion and agricultural diffuse pollution in the study area. More field work should be done in the future to corroborate the results of this study.

## Supporting Information

S1 File Soil water content data in 2012. (RAR)

S2 File Soil water content data in 2013. (RAR)

S3 File Runoff data in 2012 and 2013. (RAR)

S4 File Rainfall-runoff process data in 2012. (RAR)

S5 File Rainfall-runoff process data in 2013. (RAR)

S6 File Sediment loss data in 2012 and 2013. (RAR)
